# Advances in intestinal epithelium and gut microbiota interaction

**DOI:** 10.3389/fmicb.2025.1499202

**Published:** 2025-03-04

**Authors:** Sen Yang, Hanmin Liu, Yang Liu

**Affiliations:** ^1^Department of Pediatric Pulmonology and Immunology, West China Second University Hospital, Sichuan University, Chengdu, China; ^2^Department of Pediatrics, The Fifth Peoples Hospital of Chengdu, Chengdu, China; ^3^Key Laboratory of Birth Defects and Related Diseases of Women and Children (Sichuan University), Ministry of Education, Chengdu, China; ^4^NHC Key Laboratory of Chronobiology (Sichuan University), Chengdu, China; ^5^The Joint Laboratory for Lung Development and Related Diseases of West China Second University Hospital, Sichuan University and School of Life Sciences of Fudan University, West China Institute of Women and Children's Health, West China Second University Hospital, Sichuan University, Chengdu, China

**Keywords:** intestinal epithelium, gut microbiota, host-microbe interactions, mucosal immunity, intestinal homeostasis

## Abstract

The intestinal epithelium represents a critical interface between the host and external environment, serving as the second largest surface area in the human body after the lungs. This dynamic barrier is sustained by specialized epithelial cell types and their complex interactions with the gut microbiota. This review comprehensively examines the recent advances in understanding the bidirectional communication between intestinal epithelial cells and the microbiome. We briefly highlight the role of various intestinal epithelial cell types, such as Paneth cells, goblet cells, and enteroendocrine cells, in maintaining intestinal homeostasis and barrier function. Gut microbiota-derived metabolites, particularly short-chain fatty acids and bile acids, influence epithelial cell function and intestinal barrier integrity. Additionally, we highlight emerging evidence of the sophisticated cooperation between different epithelial cell types, with special emphasis on the interaction between tuft cells and Paneth cells in maintaining microbial balance. Understanding these complex interactions has important implications for developing targeted therapeutic strategies for various gastrointestinal disorders, including inflammatory bowel disease, metabolic disorders, and colorectal cancer.

## 1 Introduction

As a primary organ for communication between the body and the outside world, the intestine has the second largest surface area after the lungs (with the lung surface area being ~70 square meters), making it the second largest epithelial gathering place in the body (Helander and Fändriks, 2014; Derman et al., 2025). The intestinal lumen is home to a rich community of symbiotic bacteria. In addition to bacteria, archaea, fungi, viruses, and protozoa also reside in the gut (Underhill and Iliev, 2014). The intestinal epithelium is closely associated with trillions of microorganisms. Although the presence of these microorganisms is usually beneficial, the spread of gut microbes to extraintestinal organs or the overgrowth of pathogenic microbes can be disastrous for the body. On the one hand, after colonization, microbes can aid in the absorption of nutrients and play an important role in maintaining the integrity of the intestinal epithelial barrier and shaping the mucosal immune system; on the other hand, inappropriate microbial colonization can also affect host health. The ability of gut microbes to influence host health is now recognized, for example, the gut microbes colonized early in life affect children's growth and development (Robertson et al., 2019), and are associated with neonatal sepsis, neonatal necrotizing enterocolitis, childhood eczema, asthma, and diseases such as hypertension and type 2 diabetes in adulthood. In addition, some chronic gastrointestinal diseases, such as Crohn's disease, are related to the continuous immune response to gut microbes (Stappenbeck and McGovern, 2017). The intestinal epithelium, as an important part of the gut barrier, faces the huge challenge of microbes breaking through the single-layer epithelial cells of the gut to avoid abnormal immune responses. Epithelial cells construct chemical and physical barriers to isolate gut microbes from immune cells, thereby establishing a symbiotic and mutually beneficial relationship. In this review, we focus on the latest advances in the study of the interaction mechanisms between intestinal epithelium and gut microbes, understanding the close relationship between gut microbes and intestinal epithelial cells, which may promote the progress of disease diagnosis and treatment methods.

## 2 The structure and function of the intestinal epithelial barrier

The intestinal epithelium is composed of a layer of adjacent cells and intercellular junctions. In the small intestine, the epithelium extends over the structures that protrude into the lumen, forming finger-like projections (known as villi), which increase the mucosal surface area and facilitate nutrient absorption. The villi are primarily covered by absorptive columnar epithelial cells, and the spaces between the villi are the Lieber-kuhn crypts (Spence et al., 2011), which are invaginations that protect intestinal stem cells and give rise to all intestinal epithelial cell lineages. Mature intestinal epithelial cells are continuously shed into the lumen and are replaced by proliferation and differentiation of intestinal stem cells located near the base of the crypts. Under homeostatic conditions, the entire crypt is renewed approximately every 4–5 days (Moloney et al., 2016; van der Flier and Clevers, 2009). The length of the villi gradually decreases along the intestine from top to bottom, and there are no villi in the colon, resulting in a relatively flat mucosal surface that reduces potential damage caused by the passage of feces through the large intestine. The colon has expanded crypts that aid in the absorption of water and metabolic products produced by microbes (Kiela and Ghishan, 2016). Intestinal epithelial cells include a variety of different mature cell types, each with important physiological functions, including nutrient absorption (small intestine absorptive enterocytes), metabolic regulation (intestinal endocrine cells), and immune modulation (tuft cells) (Beumer and Clevers, 2016; Hooper, 2015; Kurashima and Kiyono, 2017).

**Figure 1:**
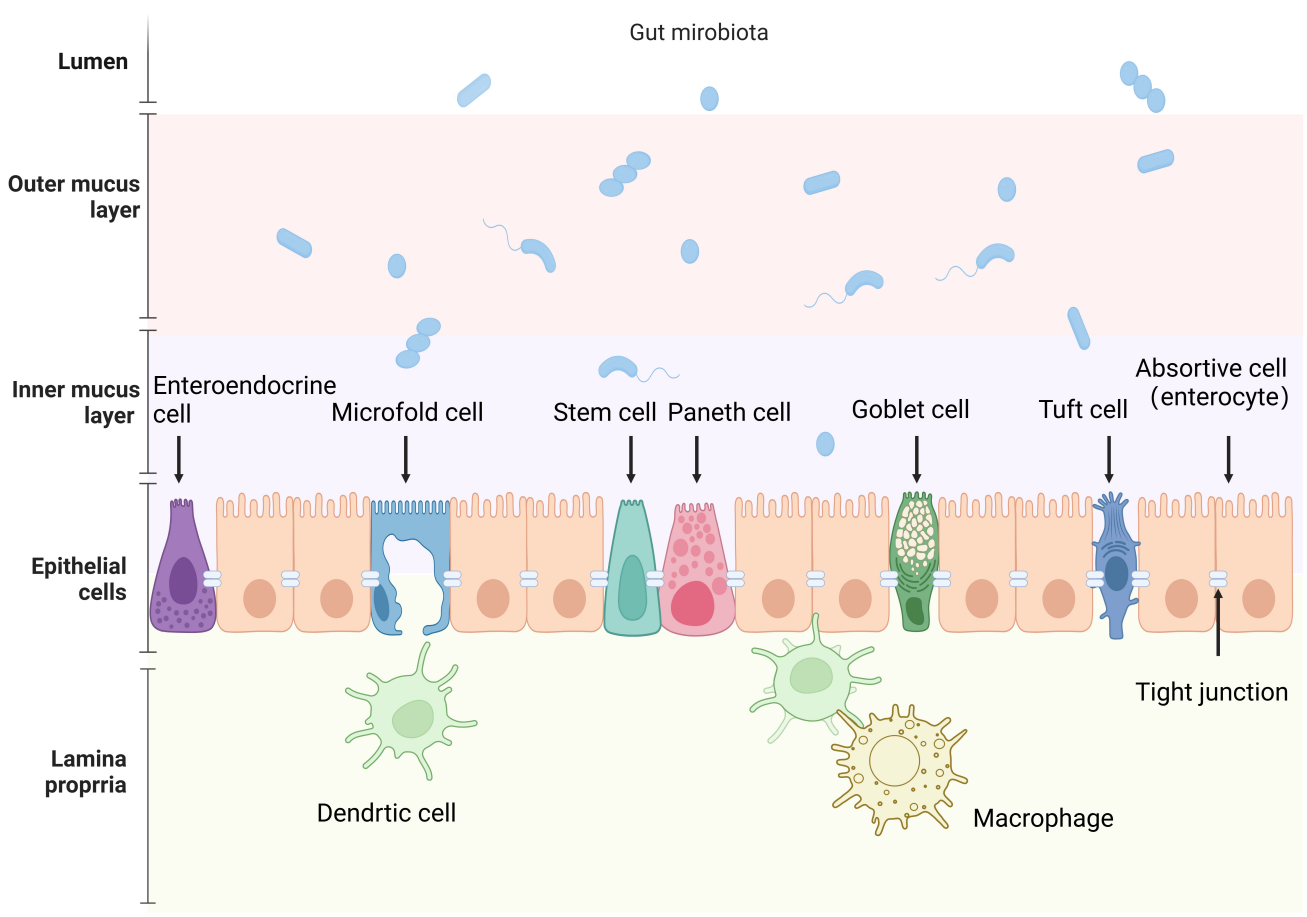
Schematic representation of the intestinal epithelial barrier and associated components. The image depicts the various cellular and structural elements that make up the intestinal barrier, from the lumen to the lamina propria. Key cell types include: absorptive cell (enterocyte), goblet cell, Paneth cell, intestinal stem cell, enteroendocrine cell, Microfold cell, and tuft cell. Created in BioRender. Liu, Y. (2025) (https://BioRender.com/m83s629).

The integrity of the intestinal epithelial barrier function requires not only a continuous cellular layer but also the composition of tight junctions between epithelial cells. These junctions serve to connect epithelial cells and regulate epithelial polarity as well as the exchange of solutes and fluids between cells (Furuse, 2010). At the same time, they physically impede microbial invasion through the paracellular route, which is of great significance for maintaining the integrity of the intestinal epithelial barrier function. Schematic diagram of the intestinal epithelial barrier can be found in Figure 1.

## 3 Characteristics of intestinal epithelial cell subtypes

The intestinal epithelium comprises various specialized cell types, each with distinct functions: Paneth cells secrete antimicrobial peptides (e.g., defensins) to regulate microbial balance, goblet cells produce mucins to forma a protective mucus layer, tuft cells detect helminths and initiate immune response; Microfold cells (M cells) transport antigens to the immune cells; enteroendocrine cells secrete hormones regulating gut physiology, and intestinal absorptive cells (enterocytes) absorb nutrients and water, respectively. Most cell types typically found in the colon are also present in the small intestine. However, certain cell types are unique to the small intestine, such as Paneth cells located at the base of the small intestinal crypts and M cells found on the follicle-associated epithelium of Peyer's patches. Table 1 provides an overview of the subtypes of intestinal cells and their main functions. As many reviews have detailed the characteristics of intestinal epithelial cells, this section will briefly summarize their key features and signaling pathways to better understand their interaction with gut microbiota.

**Table 1:** An overview of intestinal epithelial cell subtypes and their main functions.

**Type of intestinal epithelial cell**	**Distribution location**	**Introduction to functional features**	**References**
Intestinal stem cells	Small intestinal crypt, colon crypt	Replenishes the epithelial cell layer every 4–5 days	van der Flier and Clevers, 2009; Beumer and Clevers, 2016; Barker et al., 2007
Paneth cells	Small intestine	Secretes antimicrobial peptides (e.g., defensins, lysozyme); supports the stem cell niche	Clevers and Bevins, 2013; Sato et al., 2011; Ayabe et al., 2000
Goblet cells	Small intestine, colon	Secretes mucin to form a mucus barrier	Birchenough et al., 2015; Pelaseyed et al., 2014; Gustafsson and Johansson, 2022
Tuft cells	Small intestine, colon	Triggers type 2 immune response against parasites (e.g., via IL-25 secretion)	Schneider et al., 2019; Gerbe et al., 2016; von Moltke et al., 2016
M cells	Small intestine (Peyer's patches)	Transports antigens to immune cells to regulate microbial composition	Knoop et al., 2009; Kanaya et al., 2012
Enteroendocrine cells	Small intestine, colon	Secretes hormones (e.g., GLP-1, serotonin) to regulate microbial metabolism	Beumer et al., 2020; Yu et al., 2020
Absorptive cells	Small intestine, colon	Forms a physical barrier; absorbs nutrients and water; facilitates epithelial cell shedding	Kiela and Ghishan, 2016; Yin et al., 2014

### 3.1 Intestinal stem cells

Intestinal stem cell were first characterized by Cheng and Leblond (1974), identifying slender cells scattered among Paneth cells at the crypt base. These cells showed continuous cell flow from crypt to villi but not definitively proven as stem cells. Later studies confirmed the presence of stem and progenitor cells through short-lived and long-lived clones (Bjerknes and Cheng, 1999; Winton et al., 1988). LGR5+ was identified as an active stem cell marker at crypt base, while Bmi1 marks quiescent stem cells at the “+4” position (Barker et al., 2007; Potten et al., 2002). Under normal circumstances, LGR5+ cells divide rapidly for epithelial renewal (Bloemendaal et al., 2016), while “+4” position cells activated during stress to replace damaged intestinal cells (Montgomery et al., 2011; Takeda et al., 2011). The crypt balances these cell types for self-renewal and repair (Li and Clevers, 2010). Stem cells activity is regulated by multiple signaling pathways (Hou et al., 2017). WNT3, mainly produced by Paneth cells, is essential (Farin et al., 2016), with R-spondin-1 enhancing WNT signaling (Koo et al., 2012; Yan et al., 2017). Notch and BMP signaling also contribute to their proper self-renewal and differentiation (He et al., 2004).

### 3.2 Paneth cells

Paneth cells are specialized secretory cells located at the base of small intestinal crypts, interspersed among intestinal stem cells. They are characterized by large eosinophilic secretory granules containing multiple antimicrobial components such as defensins and lysozyme, which are released into the intestinal lumen to support the mucosal barrier (Clevers and Bevins, 2013). Paneth cells also play a key role in maintaining the intestinal stem cell niche (Sato et al., 2011; Clevers, 2013). They achieve this by secreting key signaling molecules such as WNT3, epidermal growth factor (EGF), and Notch ligands, which promote the proliferation and differentiation of LGR5+ stem cell. Genetic ablation of Paneth cells in mice leads to the loss of LGR5+ stem cells, highlighting their essential role in supporting the stem cell niche (Geiser et al., 2012). Abnormalities in Paneth cells are associated with many human disease processes, including Crohn's disease and graft-vs.-host disease (Eriguchi et al., 2012). WNT signaling drives Paneth cell maturation and migration to the base of the small intestinal crypts. In contrast, Notch signaling inhibits their development by suppressing ATOH1, which is critical for secretory cell differentiation (Batlle et al., 2002; Shroyer et al., 2007).

### 3.3 Goblet cells

Intestinal goblet cells are scattered among absorptive cells in the intestinal epithelium. They are characterized by a narrow cytoplasmic base and an expanded apical region filled with mucin-containing secretory granules, giving them a goblet-like appearance. These cells produce mucins, which combine with water to form mucus, creating a protective and lubricating intestinal barrier. Mucin genes are mainly divided into secreted and membrane-bound types (Dekker et al., 2002; Birchenough et al., 2015), with MUC2 being the most studied, forming a protective mucus layer (van der Post et al., 2019). Goblet cells also secrete factors like trefoil factor 3 (TFF3), which promote epithelial repair by enhancing cell migration and survival during injury (Taupin et al., 2000). In addition, goblet cells may contribute to gut homeostasis by releasing other bioactive molecules, such as antimicrobial peptides and cytokines. Their differentiation is regulated by inhibition of WNT and Notch signaling pathways.

### 3.4 Tuft cells

Tuft cells are chemosensory sentinel cells in organs such as the intestine and lungs, responding to stimuli such as hypoxia and infection (Schneider et al., 2019). They modulate mucosal immunity through G protein-coupled receptors (e.g., taste receptors). Following helminth infection and allergen deposition, the number of tuft cells and goblet cells increases rapidly. This expansion is driven by IL-4 and IL-13 secreted by type 2 innate lymphoid cells (ILC2s), which act through the STAT6 signaling pathway to promote the differentiation of tuft and goblet cells. Tuft cells further amplify this response by secreting IL-25, which enhances ILC2 activity in a positive feedback loop, aiding in innate immune responses against helminths (Gerbe et al., 2016; Sunaga et al., 2022).

### 3.5 Intestinal M cells

M cells are specialized epithelial cells that transport luminal antigens to underlying lymphoid tissues, inducing mucosal immune responses. Their differentiation relies on receptor activator of nuclear factor κB ligand (RANKL) signaling from Peyer's patch stromal cells. Experimental mice lacking RANKL signaling failed to develop M cells. RANKL induce M cell differentiation in organoids (Knoop et al., 2009) and even outside of Peyer's patches (Kanaya et al., 2012). SPIB, a transcription factor downstream of RANKL signaling (van Es et al., 2019), is essential for M cell differentiation. Its deficiency in mice results in a complete absence of M cells, impaired T cell activation, and compromised musical immune response during Salmonella infection (Kanaya et al., 2012). Enteric neurons secreting calcitonin gene-related peptide (CGRP) modulate M cell differentiation by sensing pathogens and releasing CGRP. Loss of CGRP gene eliminates the dynamic control of M cell differentiation during Salmonella infection (Lai et al., 2020).

### 3.6 Enteroendocrine cells

Enteroendocrine cells are very scarce, accounting for <1% of the total number of intestinal epithelial cells. These basal granule cells contain numerous secretory granules and exhibit neuronal-like traits, such as the ability to produce neurotransmitters and synapses-like structure (Beumer et al., 2020). The progenitors of enteroendocrine cells express neurogenin 3 (NEUROG3), which is inhibited by the Notch target HES1 (Beumer et al., 2020). NEUROG3 knockout eliminates all enteroendocrine cell subtypes in both the small and large intestine, while its overexpression increases their numbers (Li et al., 2021). The zinc finger transcription repressor GFI1 regulates secretory lineages fate, with its absence converting goblet and Paneth cells into enteroendocrine cells (Kolev and Kaestner, 2023). The differentiation of enteroendocrine cells is independent of the WNT signaling pathway, as β-catenin deletion in NEUROG3+ progenitors does not affect their development (Kolev and Kaestner, 2023; Kretzschmar and Clevers, 2017). Their differentiation requires reduced activity of the Notch and WNT pathways, a characteristic that distinguishes them from Paneth cells and absorptive cells.

### 3.7 Intestinal absorptive cells

Intestinal absorptive cells (enterocytes) are the most abundant cell type in the intestinal epithelium, responsible for absorbing ingested molecules. Notch signaling does not directly promote their differentiation but indirectly maintains their proportion by suppressing secretory lineage differentiation. When Notch signaling is inhibited in ATOH1-deficient crypts, absorptive cells develop normally and are even more abundant, likely due to reduction in secretory cell differentiation, which shifts progenitor cell fate toward the absorptive lineage (Kazanjian et al., 2010; Kim and Shivdasani, 2011). Their differentiation is further determined by the partial inhibition of WNT signaling, as demonstrated by the increased number of absorptive cells observed in organoid cultures under WNT pathway inhibition (Yin et al., 2014). Transcription factors hepatocyte nuclear factors HNF4A and HNF4G play a key role in the differentiation of absorptive cells, as knockout of HNF4G or both HNF4A and HNF4G significantly reduces absorptive cell numbers in organoids and mice (Lindeboom et al., 2018).

## 4 Influences of gut microbiota to intestinal epithelial cells

**Figure 2:**
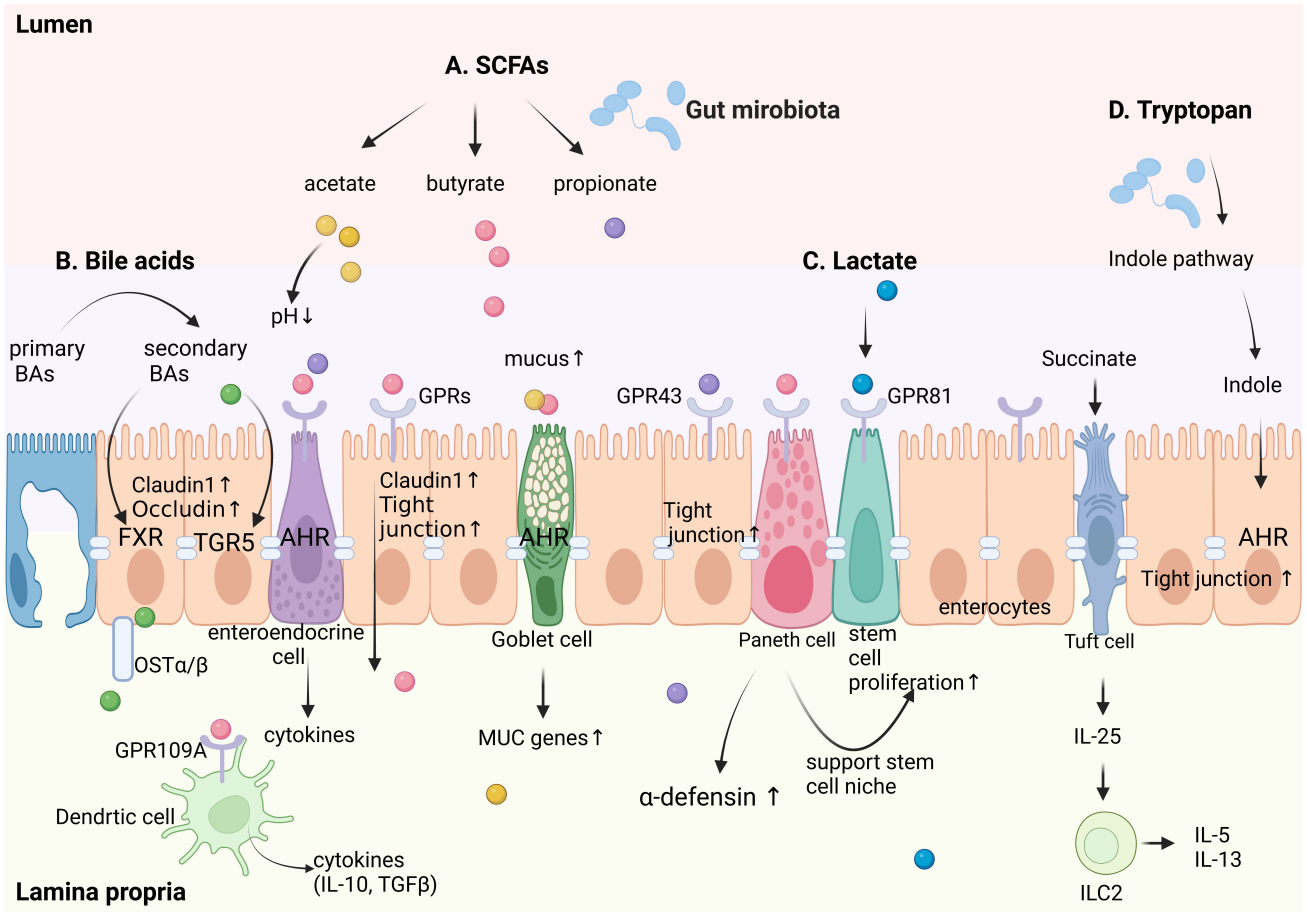
Schematic diagram of regulatory signaling pathways from intestinal microecology to intestinal epithelial cells. A. Short-chain fatty acids (SCFAs), including acetate, butyrate, and propionate, act through G-protein-coupled receptors (GPRs) such as GPR43 and GPR81 to enhance claudin-1 expression and stem cell proliferation, thereby maintaining epithelial barrier integrity. They also stimulate goblet cells to increase mucus production and Paneth cells to secrete α-defensins. B. Bile acids, converted from primary to secondary forms by gut microbes, regulate epithelial tight junction proteins (claudin-1 and occludin) via farnesoid X receptor (FXR) and TGR5 signaling. C. Lactate, activates GPR81 to support stem cell proliferation. D. Tryptophan metabolites, through the indole pathway—a hallmark microbial-mediated route in the gut—enhance tight junctions and reinforce epithelial barriers via AHR signaling. Additionally, succinate stimulates tuft cells to secrete IL-25, which activates type 2 innate lymphoid cells (ILC2) in the lamina propria, promoting the secretion of IL-5 and IL-13. Goblet cells are regulated by microbial metabolites to upregulate mucus production and MUC gene expression, strengthening the mucus barrier. Dendritic cells in the lamina propria sense microbial signals via GPR43 and produce anti-inflammatory cytokines (e.g., IL-10, TGF-β), further supporting epithelial homeostasis. This integrated network highlights the complex interplay between gut microbiota metabolites and distinct epithelial cell types, including goblet cells, Paneth cells, tuft cells, enteroendocrine cells, and enterocytes, in maintaining intestinal homeostasis. Created in BioRender. Liu, Y. (2025) (https://BioRender.com/f74q585).

Gut microbiota primarily signal to intestinal epithelial cells through metabolic products, bacterial components, and intrinsic bacterial features. The main bacterial metabolic products include short-chain fatty acids (SCFAs), lactate, bile acids (BAs), and tryptophan; bacterial components include lipopolysaccharides and flagellin, among others; intrinsic bacterial features involve bacterial adhesion, which have also been proven to play a significant role in maintaining the integrity of the intestinal epithelium (Kayama et al., 2020). Figure 2 summarized the regulatory signaling pathways from gut microbiota to intestinal epithelial cells.

### 4.1 Short-chain fatty acids and intestinal epithelial cells

In the gut, SCFAs are produced through microbial fermentation of dietary fiber, mainly as acetate, propionate, butyrate, and small amounts of valerate, caproate, and isovalerate (Koh et al., 2016). SCFAs, especially butyrate, serve not only as an energy source for the host but also as regulators of the physiological functions of intestinal epithelial and immune cells (Marchix et al., 2018). They also play an important role in maintaining the integrity of the epithelial layer and tissue repair after intestinal mucosal damage. Butyrate may enhance epithelial barrier function by upregulating Claudin-1, activating HIF-1 pathway, or promoting tight junction protein assembly by activating AMPK signaling pathways (Suzuki, 2020; Kelly et al., 2015; Hodgkinson et al., 2023). It also regulates histone acetylation by activating low concentrations of histone acetyltransferase (HAT) or inhibiting high concentrations of histone deacetylase (HDAC) classes I and II (Hodgkinson et al., 2023; Abdalkareem Jasim et al., 2022). Research has demonstrated that the effects of butyrate on cell proliferation are dose-dependent and vary across different cell types. At concentrations below 2 mM, butyrate promoted colon cell proliferation, whereas higher doses *in vitro* suppressed the growth of human colon epithelial cells (Hodgkinson et al., 2023). Oral supplementation with butyrate enhanced the villus height to crypt depth ratio in juvenile animal models, such as piglets and calves (Wang et al., 2018). Intestinal epithelial cells, especially enteroendocrine cells, express G protein-coupled receptors (GPCRs), which are crucial for immune activation and signaling molecule metabolism. SCFAs activated at least three different G protein-coupled receptors: GPR41 (free fatty acid receptor 3; FFAR3), GPR43 (free fatty acid receptor 2; FFAR2) and GPR109A (hydroxy-carboxylic acid receptor 2; HCAR2) (Parada Venegas et al., 2019). For example, GPR41/43 activation dependent on SCFAs upregulates the production of colonic epithelial cytokines and chemokines, which helps to clear pathogens (Abdalkareem Jasim et al., 2022; Hodgkinson et al., 2023; Kimura et al., 2013). Among SCFAs, butyrate primarily activates the GPR109A receptor on intestinal epithelial cells or dendritic cells in the intestinal lamina propria, while acetate and propionate exert their effects by activating the GPR43/GPR41 receptors. Table 2 briefly summarizes the main characteristics of acetate, butyrate, and propionate.

**Table 2:** The main characteristics of acetate, propionate, and butyrate.

**Function**	**Butyrate**	**Acetate**	**Propionate**	**References**
Energy source	Primary energy source for intestinal epithelial cells	Energy source for the liver and peripheral tissues	Secondary energy source metabolized in the liver	Marchix et al., 2018
Mucus secretion	Strongly promotes MUC gene expression, enhancing chemical barrier	Promotes mucus secretion, but weaker effect compared to butyrate	Promotes mucus secretion, effect between butyrate and acetate	Kimura et al., 2013
Barrier function	Upregulates Claudin-1, Occludin, and enhances tight junction proteins	Activates GPR43/GPR41, indirectly enhances barrier function	Activates GPR43/GPR41, indirectly enhances barrier function	Kelly et al., 2015; Hodgkinson et al., 2023; Kimura et al., 2013
Anti-inflammatory	Inhibits HDAC activity, directly anti-inflammatory	Regulates immunity via GPR43, indirectly anti-inflammatory	Activates GPR43, suppresses inflammatory cytokine production, indirectly anti-inflammatory	Abdalkareem Jasim et al., 2022; Kimura et al., 2013
Antimicrobial	Promotes α-defensin secretion, enhancing antimicrobial ability	Lowers pH levels, inhibiting pathogenic bacteria	Lowers pH levels, inhibiting pathogenic bacteria	Kimura et al., 2013; von Moltke et al., 2016
Immune regulation	Activates GPR109A, modulates immune cell functions	Activates GPR43, regulates dendritic cells and Tregs	Activates GPR43/GPR41, regulates dendritic cells and Tregs, promotes IL-10 secretion	Abdalkareem Jasim et al., 2022; Kimura et al., 2013; Finnie et al., 1995

Additionally, SCFAs enhance the production and release of mucus by goblet cells (Finnie et al., 1995). They specifically upregulate the expression of MUC genes in intestinal goblet cells (Fekete and Buret, 2023) and trigger Paneth cells in the small intestine to secrete α-defensin (Takakuwa et al., 2019), thereby strengthening the intestinal chemical barrier. Intestinal epithelial cells can also recognize certain byproducts such as SCFA or succinate produced by pathogens. For instance, intestinal tuft cells are chemosensors that can detect succinate produced by invading helminths. Upon activation, tuft cells trigger a type 2 innate immune pathway by producing IL-25 to clear the worms (von Moltke et al., 2016; Nadjsombati et al., 2018).

A small number of clinical trials and observational studies have found that butyrate plays a positive role in ulcerative colitis (UC) and inflammatory bowel disease (IBD). Through enema or oral administration of microencapsulated sodium butyrate, the clinical symptoms of UC or IBD patients can be improved, which may be closely related to its ability to enhance intestinal barrier function (Recharla et al., 2023; Facchin et al., 2020; Vernero et al., 2020; Scheppach et al., 1992). However, some studies have shown that butyrate enemas do not improve UC symptoms (Hamer et al., 2010). Further in-depth studies with larger sample sizes are needed.

### 4.2 Lactate and intestinal epithelial cells

Lactate is an organic acid produced by microbial fermentation of dietary fiber and other carbohydrates, such as lactose and glucose. *Bifidobacteria, Lactobacilli*, and *Enterococci* in the neonatal gut microbiota are the main producer of lactate, with some *Staphylococci* strains also capable of lactate production (Jost et al., 2012). Organoid models have confirmed that newly isolated Paneth cells from the mouse small intestine support intestinal stem cell function by providing lactic acid to enhance mitochondrial oxidative phosphorylation in Lgr5+ base columnar cells (Rodríguez-Colman et al., 2017). Recent studies have shown that lactate has unique biological activities, such as participating in the regulation of immune responses and tissue regeneration in the intestinal mucosa (Garrote et al., 2015). Lactate produced by microbial fermentation can reduce activation dependent on Toll like receptors (TLRs) and IL-1β pathways, thereby decreasing inflammatory responses in intestinal epithelial and bone marrow cells (Iraporda et al., 2015). In the Lgr5-GFP mouse model, oral administration of lactate-producing human probiotics (*Bifidobacterium* and *Lactobacillus*) significantly increased crypt height, as well as the number of Lgr5 intestinal stem cells, Paneth cells, and goblet cells in the small intestine (Lee et al., 2018). Further studies revealed that lactate signals through the G protein-coupled receptor GPR81 to induce intestinal stem cell proliferation. Additionally, pre-feeding with probiotics or lactate effectively protected mice from intestinal damage caused by radiotherapy and chemotherapy (Lee et al., 2018).

Clinical applications have also been explored, with probiotics containing *Lactobacillus acidophilus* and *Bifidobacterium bifidum* proving effective in alleviating diarrhea in cancer patients undergoing radiotherapy (Chitapanarux et al., 2010). These findings suggest that the use of *lactobacilli* symbionts or lactate salts may potentially prevent intestinal damage in humans during radiotherapy. Moreover, studies have shown that GPR81 expression is downregulated in the intestinal mucosal tissues of patients and mice with colitis. Oral administration of lactate has been found to enhance the expression of tight junction proteins, including Claudin-1, ZO-1, and Occludin, through GPR81, thereby alleviating experimental colitis and inhibiting the NF-κB/MMP9 signaling pathway (Li et al., 2024).

### 4.3 Bile acids and intestinal epithelial cells

Bile acids are synthesized from cholesterol in the liver and are mostly reabsorbed by ileal epithelial cells after entering the intestine (de Aguiar Vallim et al., 2013). Approximately 90–95% of bile acids absorbed by epithelial cells are released into the ileal lamina propria through heterodimeric organic solute transport proteins OSTα and OSTβ on the epithelial cells (Li and Chiang, 2014). In addition to playing a key role in lipid digestion and absorption, bile acids also interact with intestinal epithelial cells to maintain intestinal homeostasis, regulate immune responses, and influence the gut microbiota. They regulate the functions of intestinal epithelial cells by activating nuclear receptors such as the farnesoid X receptor (FXR) and membrane receptors like the G protein-coupled bile acid receptor TGR5 (Fiorucci et al., 2010; Dhakal and Dey, 2022). These signaling pathways not only participate in the regulation of bile acid synthesis and metabolism but also affect intestinal barrier function, cell proliferation, and apoptosis. For example, the activation of FXR can enhance the integrity of the intestinal barrier and reduce intestinal inflammation (Verbeke et al., 2015; Gadaleta et al., 2011).

Bile acids play a dual role in maintaining intestinal barrier function (Hegyi et al., 2018). Primary bile acids, including cholic acid and chenodeoxycholic acid, may exert toxic effects on intestinal epithelial cells. In contrast, secondary bile acids, such as deoxycholic acid and lithocholic acid, can enhance intestinal barrier function by regulating the expression of tight junction proteins like Claudin and Occludin, although their effects may vary under different physiological or pathological conditions (Camilleri, 2022; Di Vincenzo et al., 2022). Moreover, bile acid metabolism disorders, such as excessive accumulation of secondary bile acids or impaired reabsorption of primary bile acids, are often associated with impaired intestinal barrier function. This may lead to increased intestinal permeability and contribute to the development of IBD (Long et al., 2023).

Research on bile acid signaling pathways has introduced new strategies for treating intestinal diseases (Wahlström et al., 2016). For example, FXR and TGR5 agonists have demonstrated preclinical benefits for IBD by regulating intestinal barrier function and suppressing inflammatory responses, making them promising candidates for treating IBD and metabolic disorders (Stepanov et al., 2013). Additionally, probiotics that regulate bile acid metabolism, such as by modulating gut microbiota composition or promoting the production of secondary bile acids, have also shown potential in managing diseases like IBD (Chen et al., 2019; Gadaleta et al., 2022).

### 4.4 Tryptophan and intestinal epithelial cells

Tryptophan and its metabolites play important roles in various physiological processes, including maintaining cell growth, being a component of proteins, and coordinating the organism's response to the environment as signaling molecules (Cervenka et al., 2017). There are several metabolic pathways for tryptophan in the gastrointestinal tract: (1) the kynurenine pathway, which accounts for approximate 95% of total tryptophan metabolism; (2) the indole pathway mediated by gut microbiota, which is the characteristic metabolic pathway in the intestine; (3) the 5-hydroxytryptamine (serotonin) pathway, accounting for about 1–2% tryptophan metabolism (Ghiboub et al., 2020; Zelante et al., 2013; Yu et al., 2024). While the kynurenine pathway dominates over tryptophan metabolism systemically, the indole pathway represents the major microbial-mediated metabolic route specifically within the gut environment.

Gut microbiota metabolize tryptophan into indole and its derivatives, such as indole-3-acetic acid and indolepropionic acid. These metabolites can activate the aryl hydrocarbon receptor (AHR), which is widely present in Paneth cells, goblet cells, intestinal stem cells, absorptive cells, and enteroendocrine cells. By activating AHR, these metabolites promote the proliferation of intestinal epithelial cells and expression of tight junction proteins, maintaining the integrity of the intestinal barrier, and regulating intestinal immunity. However, excessive activation of AHR may also contribute to inflammatory responses under certain pathological conditions (Roager and Licht, 2018). Metidji's research shows that AHR regulates Wnt/β-catenin signaling in intestinal epithelial cells, which helps to differentiate epithelial cells from crypt stem cells. Meanwhile, the absence of the aryl hydrocarbon receptor in intestinal epithelial cells leads to reduced expression of MUC2 and Car4, thereby weakening resistance to pathogenic bacterial infections (Metidji et al., 2019). Some members of the human gut microbiota, such as *Clostridium sporogenes*, have been found to decarboxylate tryptophan, leading to the production of the neurotransmitter tryptamine (Williams et al., 2014). Furthermore, *Clostridium sporogenes* can lead to the production of indole acetic acid and indole propionic acid, both of which affect intestinal permeability and host immunity (Dodd et al., 2017; Lamas et al., 2018). Tryptophan and indole active transport proteins have been identified in *Escherichia coli*. These studies indicate that indole, a tryptophan metabolic product dependent on the microbiota, plays an important role in maintaining the integrity of the epithelial barrier.

### 4.5 Gut bacterial components and intestinal epithelial cells

Intestinal epithelial cells express various innate receptors, including TLRs, NOD-like receptors (NLRs), RIG-I-like receptors (RLRs), and C-type lectin receptors (CLRs). These receptors rapidly recognize microorganisms and their components (such as lipopolysaccharides, peptidoglycan, flagellin,), activate downstream signaling pathways, and subsequently promote epithelial cell proliferation as well as the expression and secretion of various cytokines and chemokines. However, excessive activation of these receptors may also lead to pathological inflammation or epithelial damage under certain conditions. This complex receptor network is essential for maintaining intestinal health and defending against pathogen invasion.

In the small intestine, Paneth cells secrete the antimicrobial peptides RegIII-β, RegIII-γ, and α-defensins in a TLR/MyD88-dependent manner under homeostatic conditions. These antimicrobial peptides play critical roles in inhibiting the proliferation of pathogenic bacteria and maintaining the balance of the intestinal microbiota (Gong et al., 2010). In the colon, goblet cells require TLR/MyD88 signaling to achieve compound mucin granule exocytosis. TLR ligands, including lipopolysaccharides and flagellin, can induce colonic goblet cells to secrete MUC2 (Birchenough et al., 2016). Studies have shown that MyD88 deficiency in intestinal epithelial cells leads to reduced expression of MUC2 and decreased production of antimicrobial peptides, particularly RegIII-γ, showing a high sensitivity to colitis and *Salmonella enterica serovar Typhi* or *Citrobacter* infection (Frantz et al., 2012; Vaishnava et al., 2011).

NLRs are innate cytoplasmic receptors that also participate in maintaining mucosal barrier function. Studies have shown that the activation of NLRP6 in the NLR family promotes the secretion of mucin granules by goblet cells, which is crucial for preventing the proliferation of colitis bacteria (such as *Prevotellaceae*) (Wlodarska et al., 2014). Intestinal endocrine cells, Paneth cells, and goblet cells can specifically express Chitinase 3-like protein 1 (Chi3l1) and secrete it into the intestinal lumen when stimulated by the gut microbiota. Chi3l1 interacts with the gut microbiota through the cell wall component peptidoglycan, affecting the colonization of Gram-positive bacteria. This interaction not only prevents colitis but also contributes to the regulation of immune responses and the maintenance of the intestinal barrier (Chen et al., 2024).

### 4.6 Bacterial intrinsic features and intestinal epithelial cells

Some commensal bacteria have evolved specific strategies that allow them to adhere to the intestinal mucosal surface and induce the expression of specific genes in intestinal epithelial cells, which is related to the intrinsic features of the bacteria.

Segmented filamentous bacteria (SFB) are natural gut commensals. Through comparative studies in humans, mice, and chickens, it has been found that while SFB distribution in the gastrointestinal tract shows species specificity, the small intestine (particularly the ileum) serves as the primary colonization site across all studied species (Yin et al., 2013). SFB communicates with host ileal epithelial cells through endocytic vesicles formed at the SFB-epithelial cell synaptic interface. These vesicles contain SFB cell wall-associated proteins P3340 that can induce the activation of antigen-specific Th17 cells in the lamina propria by promoting antigen presentation. This confirms direct communication between resident gut microbiota and the host, and indicates that under physiological conditions, intestinal epithelial cells acquire antigens from commensal bacteria to generate T cell responses to the resident microbiota (Ladinsky et al., 2019; Yang et al., 2014). SFB colonization in the small intestine promotes overall transcriptional changes in host epithelial cells, including the induction of antimicrobial peptides and stress response genes, such as serum amyloid A (SAA1 and SAA2) (Ivanov et al., 2009).

Unlike SFB, which colonizes the small intestine, *Bacteroides* predominantly resides in the colonic crypts. *Bacteroides* plays a crucial role in maintaining gut microbiota balance by fermenting polysaccharides to produce short-chain fatty acids. Studies (Lee et al., 2013) in germ-free mice showed that animals were easily colonized first by *Bacteroides fragilis*, followed by *Bacteroides thetaiotaomicron* or *Bacteroides vulgatus*, with the sequence of microbial exposure having no effect on colonization results. Further investigation showed that intestinal *Bacteroides* possess conserved polysaccharide utilization loci, known as commensal colonization factors (CCF). During intestinal colonization, the CCF gene in *Bacteroides fragilis* are upregulated. Deletion of the CCF gene in the symbiont *Bacteroides fragilis* led to colonization defects and reduced horizontal transmission in mice. Notably, mutant strains lacking CCF failed to penetrate deep into the colonic crypts despite binding to the epithelial surface. These findings demonstrate that intestinal *Bacteroides* have developed unique, host-specific interactions that ensure stable and resilient gut colonization, with the CCF serving as an innovative mechanism driving this symbiotic relationship (Lee et al., 2013).

The intrinsic features of commensal bacteria play a crucial role in their ability to interact with intestinal epithelial cells and establish stable colonization. Understanding these interactions provides valuable insights into the dynamic relationship between gut microbes and their host, offering potential avenues for advancing gut health research.

## 5 Regulation of gut microbiota by intestinal epithelial cells

The mucosal barrier system of the intestine includes physical and chemical barriers. The physical barrier consists of the mucus layer, a protective layer formed by carbohydrates on the cell membrane surface, and the intercellular junction layer (Pelaseyed et al., 2014). The chemical barrier is primarily composed of antimicrobial peptides, the Reg3 family of proteins, and other secreted substances within the mucus layer that covers intestinal cells (Vaishnava et al., 2011; Ayabe et al., 2000; Mukherjee and Hooper, 2015).

Furthermore, the initiation of inflammatory cascade reactions in intestinal epithelial cells can lead to antimicrobial responses, thereby regulating bacterial composition. Paneth cells have a significant impact on the microbial composition in the small intestine by secreting various antimicrobial peptides, such as α-defensins and Reg3 proteins, which regulate bacterial colonization. Changes in the chemical and genetic characteristics of Paneth cells in mice can lead to significant and lasting changes in the microbiome, including a substantial reduction in *Proteobacteria* (Lueschow et al., 2018; Gassler, 2017). Among these, α-defensins protect the host from pathogenic bacterial infections by disrupting bacterial cell membrane (Selsted and Ouellette, 2005). Studies have shown that antimicrobial peptides produced by human α-defensin 5 (HD5) have direct bactericidal effects on several members of the human microbiome, thus altering the bacterial community within the body (Ehmann et al., 2019). Researchers compared the gut microbiota of mice expressing human α-defensin 5 with that of mice lacking the enzyme required for processing α-defensins and found significant α-defensin-dependent changes in the composition of the microbiota (Salzman et al., 2010). Moreover, HD5 transgenic mice were resistant to *Salmonella* infection (Salzman et al., 2003). In addition, the Reg3 family of proteins, mainly produced by Paneth cells, exhibit bactericidal activity against Gram-positive bacteria (Cash et al., 2006). Multiple studies have shown that Paneth cell dysfunction is closely associated with Crohn's disease, which may also be associated with disruptions in gut microecology (Adolph et al., 2013).

Mucins secreted by goblet cells form mucus with water, which serves as a medium for the colonization of commensal gut microbes and a protective barrier against pathogenic bacteria, thereby maintaining immune homeostasis (Yang and Yu, 2021; Gustafsson and Johansson, 2022). The highly glycosylated mucins that make up the main structural components of the mucus layer are a primary carbon source for certain gut microbes, such as *Akkermansia muciniphila*, whose abundance is inversely proportional to the severity of inflammatory diseases (Derrien et al., 2004). Therefore, mucins remain an important way for goblet cells to nourish microbes and regulate the microbial composition structure through this strategy.

Tuft cells play a complex role in regulating gut microbes through multiple mechanisms. They indirectly regulate gut microbes during type 2 immune responses. The process involves triggering ILC2 to release IL-13, which allows tuft cells to interact with IL-13-responsive goblet cells to release mucus, capable of clearing both eukaryotic and bacterial pathogens. In the small intestine, tuft cells differentially express succinate receptor 1, allowing intestinal protozoan monocytes and certain bacteria that produce succinate to activate corresponding signaling pathways, thereby regulating the gut microbiota (Schneider et al., 2019). Additionally, tuft cells sense bacterial metabolite N-undecanoyl glycine (N-C11-G) via the vomeronasal receptor Vmn2r26, activating the production of prostaglandin D2, which in turn stimulate goblet cells to secrete mucus and initiates antibacterial immune response (Coutry et al., 2024). Uniquely, tuft cells are the only known intestinal epithelial cells to express choline acetyltransferase (ChAT), the enzyme essential for acetylcholine biosynthesis. Recent studies have shown that tuft cell-derived acetylcholine plays a pivotal role in clearing worm infections (Billipp et al., 2024). Furthermore, latest research highlights the ability of tuft cells to maintain intestinal microecological balance with Paneth cells, underscoring their significant role in regulating gut homeostasis (Coutry et al., 2023).

As the primary epithelial medium for antigen uptake, M cells are also crucial for regulating gut microbes. Studies have shown that the transient depletion of M cells leads to an increase in the levels of ileal SFB, indicating that M cells can regulate the abundance of ileal SFB (Lai et al., 2020).

As previously mentioned, gut endocrine cells express multiple G-coupled receptors and toll-like receptors, which can, respectively, recognize SCFA and respond to TLR ligands of gut bacteria (Yu et al., 2020). In addition, enteroendocrine cells synthesize and release serotonin (5-HT), the number of which and the synthesis and release of 5-HT are regulated by microbes, parasites, and immune cells (Mawe and Hoffman, 2013). Research suggests that during intestinal infections, the production of 5-HT helps prevent the invasion of microbes (such as *Salmonella typhimurium*) and helminth (*Trichomonad*) pathogens, possibly by regulating the functions of intestinal cells. This includes enhancing antimicrobial peptide secretion and modulating IL-13 receptor signaling pathway (Essien et al., 2013).

Intestinal stem cells are located at the base of the small intestinal crypts. Although most gut microbes reside in the mucosal layer above the villi, a subset known as the crypt-restricted core microbiome remains within the crypts, adjacent to the stem cell niche (Hou et al., 2017).

In summary, various intestinal epithelial cell subtypes exert regulatory effects on the microbiome (as shown in Table 3). While most of the microbiome effects of individual intestinal epithelial cell subtypes have been extensively studied, understanding how these cell subtypes communicate with each other to coordinate their response to the microbiome warrants further investigation.

**Table 3:** A summary of mechanisms by which intestinal epithelial cells regulate gut microbiota.

**Intestinal epithelial cell subtype**	**Main function**	**Mechanisms for regulating gut microbiota**	**References**
Paneth cells	Secrete antimicrobial peptides (e.g., defensins)	Directly inhibit the growth of harmful bacteria and maintain microbial balance by secreting antimicrobial peptides (e.g., α-defensins and lysozyme)	Clevers and Bevins, 2013; Sato et al., 2011
Goblet cells	Secrete mucus to protect the barrier	Form a physical barrier by secreting mucus, isolating the microbiota from epithelial cells to prevent excessive contact	Birchenough et al., 2015; Pelaseyed et al., 2014
Enteroendocrine cells	Secrete hormones (e.g., serotonin, GLP-1)	Regulate gut microbiota metabolism by secreting hormones; some hormones (e.g., serotonin) influence microbiota composition and activity	Lai et al., 2020; Yu et al., 2020; Mawe and Hoffman, 2013
Tuft cells	Sense microbial metabolites	Detect microbial metabolites (e.g., N-C11-G) and activate immune responses, indirectly promoting microbiota balance	Schneider et al., 2019; Billipp et al., 2024
M cells	Antigen sampling	Transport antigens to immune cells and regulate the abundance of specific microbiota (e.g., modulating ileal SFB levels)	Knoop et al., 2009; Kanaya et al., 2012; Coutry et al., 2023
Enterocytes	Nutrient absorption	Regulate microbiota metabolism and composition indirectly by absorbing metabolites such as short-chain fatty acids (SCFAs)	Kiela and Ghishan, 2016; Hou et al., 2017
Intestinal stem cells	Maintain epithelial renewal	Influence the microenvironment by secreting signaling molecules (e.g., Wnt and Notch), indirectly regulating microbiota composition	van der Flier and Clevers, 2009; Farin et al., 2016

SCFAs, short-chain fatty acids; SFB, segmented filamentous bacteria; GLP-1, glucagon-like peptide-1.

## 6 Conclusion and future perspectives

The complex interplay between the intestinal epithelium and gut microbiota is a cornerstone of maintaining intestinal homeostasis and overall health. This review highlights recent advances in understanding the bidirectional communication between intestinal epithelial cells and microbial communities, emphasizing the specialized roles of epithelial cell subtypes such as Paneth cells, goblet cells, tuft cells, and enteroendocrine cells. These cells not only form a physical and chemical barrier but also actively participate in regulating microbial composition, immune responses, and metabolic functions.

Emerging evidence underscores the importance of gut microbiota-derived metabolites, such as short-chain fatty acids and bile acids, in modulating epithelial cell signaling pathways and maintaining barrier integrity. Furthermore, the cooperative interactions among epithelial cell subtypes, particularly between tuft cells and Paneth cells, reveal a sophisticated network that ensures microbial balance and host defense. These findings offer new insights into the dynamic and reciprocal nature of host-microbe interactions.

Looking forward, several key areas warrant further exploration. First, the application of single-cell sequencing and organoid models holds great promise for uncovering previously unrecognized mechanisms of epithelial-microbiota crosstalk. Second, understanding how disruptions in these interactions contribute to the pathogenesis of diseases, such as inflammatory bowel disease, colorectal cancer, and metabolic disorders, remains a critical challenge. Finally, translating these findings into targeted therapeutic strategies, such as microbiota-based interventions or epithelial cell-specific therapies, represents an exciting frontier in precision medicine.

In conclusion, advancing our understanding of the intestinal epithelium-microbiota axis will not only deepen our knowledge of gut physiology but also pave the way for innovative approaches to treat and prevent a wide range of gastrointestinal and systemic diseases.
